# Cationic *N*,*N*-Dimethylglycine Ester Prodrug of 2*R*-α-Tocotrienol Promotes Intestinal Absorption via Efficient Self-Micellization with Intrinsic Bile Acid Anion

**DOI:** 10.3390/molecules27092727

**Published:** 2022-04-23

**Authors:** Daisuke Watase, Shuichi Setoguchi, Nami Nagata-Akaho, Shotaro Goto, Hirofumi Yamakawa, Ayano Yamada, Mitsuhisa Koga, Yoshiharu Karube, Kazuhisa Matsunaga, Jiro Takata

**Affiliations:** Faculty of Pharmaceutical Sciences, Fukuoka University, Nanakuma, Jonan-ku, Fukuoka 814-0180, Japan; watase@fukuoka-u.ac.jp (D.W.); ssetoguchi@fukuoka-u.ac.jp (S.S.); nanohana_73@jewel.ocn.ne.jp (N.N.-A.); sgoto@fukuoka-u.ac.jp (S.G.); hyamakawa@adm.fukuoka-u.ac.jp (H.Y.); a.yamada.or@adm.fukuoka-u.ac.jp (A.Y.); kogami@fukuoka-u.ac.jp (M.K.); karube@fukuoka-u.ac.jp (Y.K.); jtakata@fukuoka-u.ac.jp (J.T.)

**Keywords:** bile acid, bioavailability, drug delivery system, micelle, prodrug, tocotrienol, vitamin E

## Abstract

The intestinal absorption of hydrophobic compounds is severely influenced by their transportation rate through the unstirred water layer in the intestinal lumen. A member of the vitamin E family, α-Tocotrienol (α-T3) has remarkable pharmacological effects, but its intestinal absorption is hampered due to its hydrophobicity. Here, we prepared three ester derivatives of 2*R*-α-T3, and we selected a suitable prodrug compound using rat plasma and liver microsomes. The micellization profile of the selected compound in the presence of taurocholic acid (TCA) was evaluated. After gastrostomy administration of the prodrug candidate or α-T3 solution containing TCA, *AUC* values were determined for α-T3 in plasma obtained from bile duct-ligated rats. Among the three types in the efficiency of the reconversion to the parent drug, α-T3 *N*,*N*-dimethylglycinate (α-T3DMG) was the best prodrug; α-T3DMG formed mixed micelles via ion pairs with anionic TCA. The solubility of α-T3DMG in n-octanol/water depended on its ratio to TCA. The *AUC* after α-T3DMG administration to ligated rats was 2-fold higher than that after α-T3 administration, suggesting a smooth interaction with intrinsic bile acids. In conclusion, utilization of the prodrug synthesized using *N*,*N*-dimethylglycine ester may be a beneficial approach to promote intestinal absorption of α-T3 via self-micellization with intrinsic bile acid.

## 1. Introduction

Oral administration is the preferred route of medication due to moderate emergence of the effect, high convenience, and patient adherence in terms of ease in use and portability, as well as lower manufacturing costs. However, an active compound in the oral dosage form must undergo degradation, complexation, and/or precipitation in the gastrointestinal tract. In addition, the compound must pass through the barrier at the intestinal membrane and survive first-pass metabolism. To achieve good bioavailability, it is believed that low-molecular-weight compounds must have adequate hydrophilicity to dissolve in the unstirred water layer in the intestinal lumen and simultaneously have sufficient hydrophobicity to permeate across the apical cell membrane, which is often a trade-off problem. Therefore, obtaining sufficient intestinal absorption through oral administration is one of the most challenging issues in the development of medicine [1,2].

**Figure 1:**
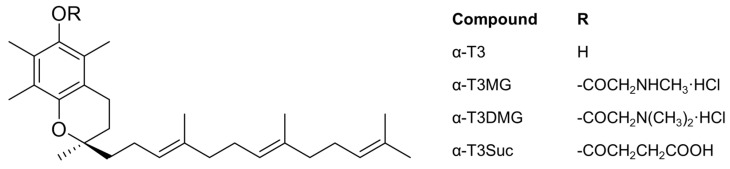
Chemical structures of 2*R*-α-Tocotrienol (α-T3) derivatives.

A member of the vitamin E family, α-Tocotorienol (α-T3) is different from α-tocopherol—a well-known representative player in the family—in that the side chain is saturated (Figure 1) [3]. It has been reported that α-T3 has a variety of pharmacological effects and a high membrane permeability, which is independent of tocopherols [4]; thus, it may be a good candidate for use as a neuroprotective agent [5,6,7], a radioprotector [8,9], and an anticancer agent [10,11]. However, intestinal absorption of the lipid vitamin E family is quite low, which is a critical step in obtaining sufficient bioavailability [12,13,14,15,16]. In the intestinal lumen, bile acid/salt and phospholipids are secreted, facilitating the solubilization and the emulsifying of lipid food ingredients/drug compounds to permeate through the unstirred water layer [1,2]. Therefore, sufficient intestinal absorption depends on whether a hydrophobic compound can be micellized/solubilized by intrinsic bile. An innovative self-emulsified drug delivery system (SEDDS), such as a cyclosporin SEDDS, is effective in improving low oral bioavailability [13,17]. However, this formulation technique requires larger dosage volumes because it requires a high amount of fatty acid triglycerides and surfactants in the dosage regimen.

We previously reported prodrug strategies for the intravenous administration of vitamin E (γ-tocopherol and γ-tocotrienol) and reduced vitamin K (active form) [18,19,20,21]. We also proposed prodrugs of reduced coenzyme Q_10_ for oral administration, which can formulate smaller dosage volumes that form very small particles (~5 nm) with endogenous bile acids [22]. This is an improved procedure for the development of suitable dosage formulations for easier oral uptake and enhanced intestinal diffusion.

In this study, to establish a novel prodrug of α-T3 for oral administration, we prepared three ester derivatives of α-T3 (Figure 1), and we evaluated them as prodrug candidates using rat plasma and microsomes. After selecting the best prodrug, we investigated the complexation in vitro between the α-T3 cationic prodrug and taurocholic acid (TCA), a major bile acid. To elucidate the influence of bile acid on intestinal absorption of α-T3 using its prodrug, plasma concentrations of α-T3 with TCA after gastrostomy administration of the prodrug to a bile duct-ligated rat model were determined.

## 2. Results

### 2.1. Physicochemical Properties of α-T3 Ester Derivatives

Three ester derivatives of α-T3 were successfully obtained (Figure 1) and characterized by mass spectrometry (MS), elemental analysis, and ^1^H-^13^C heteronuclear multiple-bond correlation nuclear magnetic resonance (NMR). All three ester derivatives were stable to oxidation under atmospheric conditions. The water solubility of α-T3MG and α-T3DMG was greater than 50 mmol/L; α-T3DMG did not exhibit any degradation in water when incubated at 5 and 20 °C for 28 days. Under an accelerated condition at 40 °C, the half-life of α-T3DMG was approximately 27 days.

### 2.2. Enzymatic Hydrolysis of α-T3 Ester Prodrug Candidates

To verify whether α-T3 ester derivatives can be activated by esterase and regenerate to the parent form (α-T3), hydrolysis of rat plasma and liver microsomes was assessed. The velocity versus concentration profile was fitted using the Michaelis–Menten model, and the kinetic parameters were calculated using GraphPad Prism (Table 1). Hydrolysis of α-T3 ester derivatives was accelerated in both rat plasma and liver microsomes. Hydrolysis efficiency *is V*_max_/*K*_m_ is. The *V*_max_/*K*_m_ values of α-T3 ester derivatives in plasma were similar to each other at approximately 0.1, whereas those in the liver microsomes were clearly different, and the order was α-T3DMG > α-T3MG > α-T3Suc. This result indicated that α-T3DMG was the best prodrug candidate among the three compounds. Furthermore, to clarify whether the hydrolysis of α-T3DMG and α-T3MG can be inhibited by eserine, an esterase inhibitor, α-T3DMG, and α-T3MG hydrolysis was measured in rat liver microsomes. As shown in Supplementary Figure S1, eserine inhibited both α-T3DMG and α-T3MG hydrolysis in a concentration-dependent manner. Based on this comparison, only α-T3DMG was subjected to the following experiments.

### 2.3. Micellization Profile of α-T3DMG with TCA

**Figure 2:**
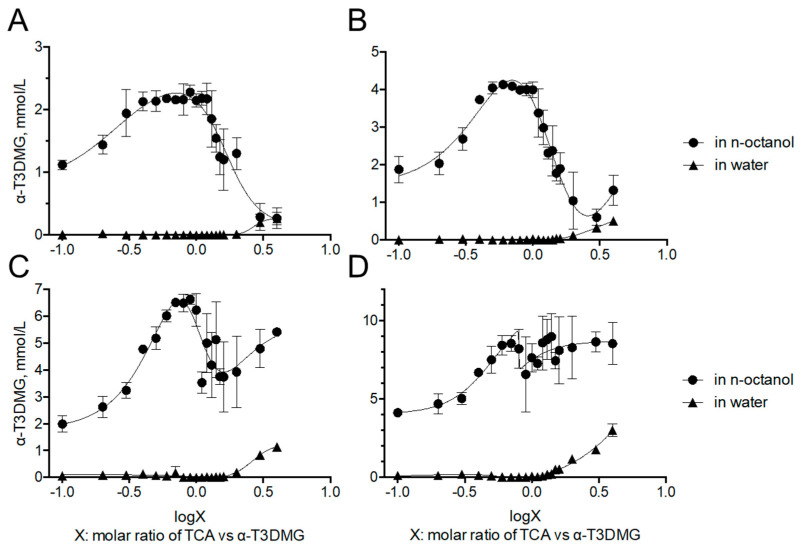
Distribution of α-T3DMG to n-octanol and water phases. α-T3DMG concentrations in the test solutions are (**A**) 2.94 mmol/L, (**B**) 5.88 mmol/L, (**C**) 11.76 mmol/L, and (**D**) 23.52 mmol/L. The values indicate mean ± SD (*n* = 3). The actual concentrations of TCA are summarized in Table 4 in the method Section 4.7.1.

**Figure 3:**
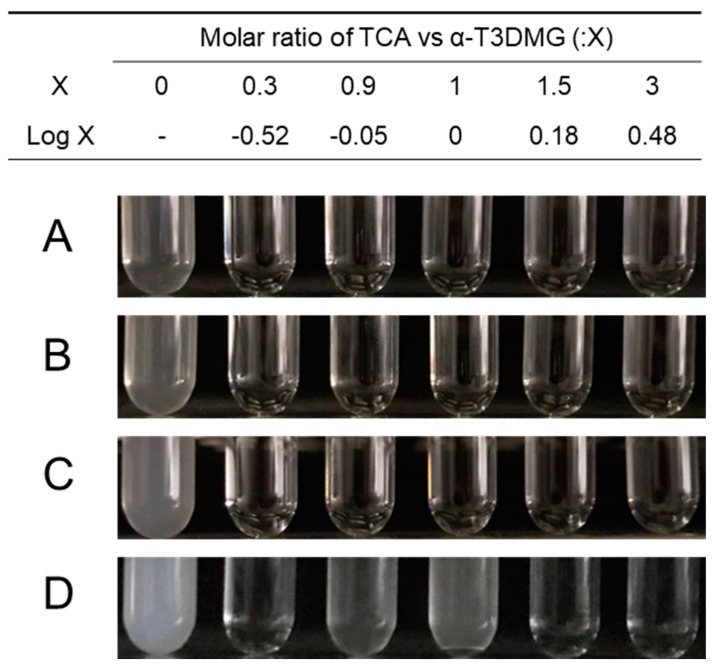
Appearance of aqueous solutions of α-T3DMG with or without TCA. α-T3DMG concentrations in the test solutions are (**A**) 2.94 mmol/L, (**B**) 5.88 mmol/L, (**C**) 11.76 mmol/L, and (**D**) 23.52 mmol/L.

**Figure 4:**
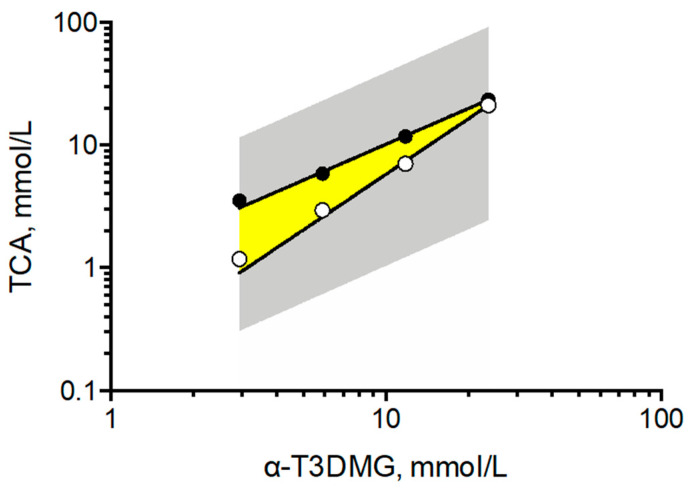
Phase diagram of solubility of α-T3DMG in the presence of TCA. The gray area covers the points examined in this study. The yellow area indicates ion-pair complex between α-T3DMG and TCA, obtained from the data as solubility saturation of α-T3DMG in octanol phase or precipitation in water in Figure 2 and Figure 3.

To clarify the interaction between α-T3DMG and taurocholic acid (TCA), a representative bile acid was investigated (Figure 2, Figure 3 and Figure 4, Table 2). In the absence of TCA, the appearance of α-T3DMG in water was cloudy at all concentrations (2.94–23.52 mmol/L), as shown in Figure 3. The particle sizes were in the submicron order (Table 2). In the presence of TCA, the appearance of α-T3DMG became transparent, except for the mixtures of 23.52 mmol/L α-T3DMG with TCA for α-T3DMG:TCA molar ratios of 1:0.9 and 1:1 (Figure 3D). Although the z-averages and the polydispersity index (PDI) were ambiguous because of rapid sedimentation (Table 2) at a ratio of 1:1, the particle size was approximately 3200 nm, reflecting the precipitation in Figure 3D. The α-T3DMG levels in the n-octanol phase increased at lower molar ratios of TCA versus α-T3DMG and reached a plateau at approximately equal molar ratios of α-T3DMG and TCA (Figure 2). At TCA concentrations over the plateau, the α-T3DMG levels in the n-octanol phase decreased gradually or remained constant. Simultaneously, the α-T3DMG levels in the water phase increased in a TCA-concentration manner. A phase diagram of these results is shown in Figure 4. The initial positive z-potential of the test solution decreased with the addition of TCA, and over the ratio of α-T3DMG:TCA = 1:1.5, the potentials reversed to a negative charge (Table 2). The z-potential of the TCA without α-T3DMG in water (23.52 mmol/L) was −38.9 ± 1.9 mV.

### 2.4. Influence of TCA on Intestinal Absorption of α-T3 after Administration of α-T3DMG

**Figure 5:**
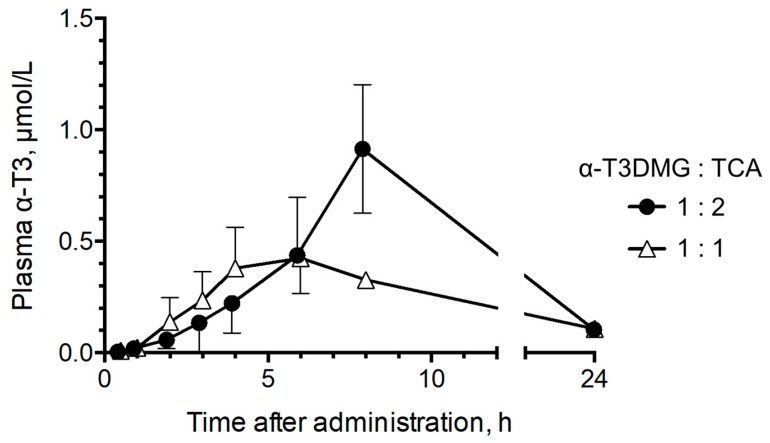
Plasma concentration of α-T3 after gastrostomy administration of α-T3DMG with TCA in bile duct-ligated rats. Closed circle, 5.88 mmol/L α-T3DMG and 5.88 mmol/L TCA in water (α-T3DMG:TCA = 1:1); open triangle, 5.88 mmol/L α-T3DMG and 11.76 mmol/L TCA in water (α-T3DMG:TCA = 1:2). The doses are 12.5 mg/kg of weight equivalent for α-T3. The values indicate mean ± SD (n = 3).

To confirm the influence of the interaction with TCA on the intestinal absorption of α-T3 using α-T3DMG, the plasma concentrations in bile duct-ligated rats that were administered a mixture of α-T3DMG and TCA via gastrostomy were determined (Figure 5). The pharmacokinetic parameters are listed in Table 3. The C_max_ and the AUC of plasma levels of α-T3 after the administration of solubilized micelles (α-T3DMG:TCA = 1:2) were approximately 2-fold higher than those after ion-pair administration (α-T3DMG:TCA = 1:1). The plasma levels of α-T3DMG (ester form) were 4–10% of that of α-T3.

## 3. Discussion

To achieve sufficient intestinal absorption, drug solubility in the unstirred water layer is essential [1,2]. The SEDDS strategy promotes the solubility of hydrophobic compounds in the water layer, inducing intrinsic bile flow by large volumes of lipids and surfactants [13,17]. However, this strategy is not a compact dosage form. A member of the vitamin E family, α-T3 has unique phamacological effects that are independent of tocopherols’ [4,5,7,10]. It is well-known that fat soluble vitamins also have low intestinal absorption, and the absorption depends on solubilization with bile acid [12,13,14]. In the present study, we aimed to establish a novel ionic prodrug of water-insoluble α-T3 and to clarify whether the prodrug could self-solubilize with intrinsic bile acid in the intestinal lumen and enhance the intestinal absorption of α-T3.

The three obtained ionic esters of α-T3 were stable against oxidation. Cationic α-T3MG and α-T3DMG were obtained as solid powders with high melting points. Cationic ester derivatizations improved the water solubility/dispersity of α-T3; α-T3DMG in water was stable at room temperature (20 °C) for a month. These physicochemical properties have some merit for the development of a variety of dosage forms, such as tablets and injection dosage forms.

The ester-type prodrug needs to be reconverted to its parent form via esterase at a target site. To clarify the function of these three prodrug candidates, a hydrolysis study to the parent compound (α-T3) was performed. The V_max_/ K_m_ values in rat plasma, which indicate the rates of hydrolysis, were not different among the three compounds. In contrast, that of α-T3DMG in rat liver microsomes, which includes a rich amount of esterase, was much higher than that of the other two ester compounds. Therefore, the reconversion rate of α-T3DMG to its parent form was the highest among the three candidates. In addition, hydrolysis of α-T3DMG and α-T3MG using rat liver microsomes was completely inhibited by eserine (Supplementary Figure S1). These results showed that α-T3DMG could act as a good prodrug for liver esterase.

To achieve sufficient solubility, bile acids/salts and phospholipids in bile solubilize hydrophobic compounds facilitate transport through the unstirred water layer. Thus, it is important to verify the solubility of the compounds of interest to estimate intestinal absorption and to understand the absorption mechanism. The cationic prodrug α-T3DMG selected for the hydrolysis study was expected to form a complex with anionic bile acid. As a representative bile acid, the interaction between TCA and α-T3DMG was investigated in vitro. For the changes in the appearance of mixed solutions of α-T3DMG with TCA, white precipitation was observed at an equal molar ratio of TCA versus 23.52 mmol/L α-T3DMG, which is the highest concentration used in this study. The occurrence of the ion-pair coacervation can be explained by α-T3DMG and TCA having single valencies of cations and anions, respectively. The lower concentrations of α-T3DMG (2.94, 5.88, and 11.76 mmol/L) did not cause any precipitation. At no less than 1.5-times ratios of TCA versus α-T3DMG, the distribution of α-T3DMG to n-octanol decreased and that to water increased. Based on the results of the zeta-potentials of the mixture solutions, it is speculated that adding anionic TCA to cationic α-T3DMG initially forms α-T3DMG-rich mixed micelles and then ion-pair micelles that are non-polar complexes, and an increase in their distribution to n-octanol is formed at equal molar ratios between TCA and α-T3DMG. At excess molar ratios of TCA versus α-T3DMG, TCA-rich mixed micelles were formed, and their distribution to n-octanol decreased. In addition to the component with more TCA, it is speculated that the ion-pair complex is solubilized by TCA micelles, and the micelles become amphiphilic to both the n-octanol and the water layers.

To confirm the influence of TCA on α-T3 absorption using α-T3DMG, bile duct-ligated rats were administered 5.88 mmol/L α-T3DMG in water in the presence of TCA via the gastrostomy route. The doses were 12.5 mg/kg of weight equivalent for α-T3. The amount of bile acid secreted in the fasting and in the postprandial states was approximately 5 and 10 mmol/L, respectively [1]. Considering the bile levels in the intestine, TCA doses were set as 5.88 and 11.76 mmol/L (molar ratios 1 and 2 versus α-T3DMG), reflecting fasted and postprandial states, respectively. The plasma α-T3 levels after postprandial administration of α-T3DMG were 2-fold higher than those in fasted rats. Furthermore, the plasma α-T3 levels after postprandial administration of α-T3 were similar to those after fasting administration of α-T3DMG (Supplementary Figure S2). These results indicate that α-T3DMG can form mixed micelles efficiently and enhance the intestinal absorption of α-T3. The ester bond in α-T3DMG would be rapidly cleaved by esterases in the intestinal epithelial cells and hepatic parenchymal cells; α-T3 (parent form) will then enter the systemic circulation. The plasma levels of α-T3DMG were much lower than that of α-T3. It is well-known that elimination of α-T3 is faster than tocopherols. In addition, α-T3 is a safe compound which is present in natural foods such as palm oils and rice oils. In the present in vivo study, no toxicity or abnormalities were observed in rats and in blood samples. Therefore, α-T3DMG could be a highly safe molecule for clinical use through oral delivery.

In conclusion, α-T3DMG can form mixed micelles at the intrinsic bile acid level in the postprandial state, improving the water solubility and the intestinal absorption of α-T3. As α-T3DMG does not require any other additives such as fatty acids and surfactants that tend to induce bile secretion, this prodrug strategy can be used as a compact self-micellization dosage form. Further studies for detailed physicochemical properties and pharmacokinetic profiles using artificial human bile components and rat models for comparison with SEDDS are required.

## 4. Materials and Methods

### 4.1. Chemicals

Tocotrienol-rich fraction of palm oil was purchased from Tama Biochemical Co., Ltd. (Tokyo, Japan). Using normal-phase chromatography, α-T3 was isolated from the fraction. *N*-*t*-Boc-*N*-methylglycine hydrochloride was purchased from the Peptide Institute, Inc. (Osaka, Japan), and *N*,*N*-dimethylglycine hydrochloride was purchased from Tokyo Kasei Kogyo Co., Ltd. (Tokyo, Japan). All other chemicals were purchased from FUJIFILM Wako Pure Chemical Corporation (Osaka, Japan).

### 4.2. Animals

Male Sprague–Dawley (SD) rats (8 weeks old) and SD rat liver microsomes were obtained from the Jackson Laboratory, Japan, Inc. (Kanagawa, Japan). All animal care and use procedures were performed in compliance with the regulations established by the Experimental Animal Care and Use Committee of Fukuoka University (approved number: 1407756), which are in accordance with the universal principles of laboratory animal care.

### 4.3. Instrumental Analysis

All melting points were determined using a BY-1 micro-melting point apparatus (Yazawa, Tokyo, Japan), and they were uncorrected. Elementary analyses and ^1^H-NMR and mass spectra measurements were performed at the Central Microanalytical Department of Pharmaceutical Sciences, Fukuoka University. Using a JEOL GX-500b spectrometer (JEOL Ltd., Tokyo, Japan), ^1^H-NMR spectra were recorded at 500 MHz in CDCl_3_. The chemical shifts were expressed in δ (ppm), using tetramethylsilane as the internal standard, with the following abbreviations: s = singlet, d = doublet, m = multiplet. The coupling constant J was measured in Hz. Fast-atom bombardment mass spectra were obtained using a JEOL DX-300 spectrometer (JEOL Ltd., Tokyo, Japan).

### 4.4. Synthesis of α-T3 Ester Derivatives

#### 4.4.1. R-α-Tocotrienyl *N*-Methylglycinate Hydrochloride

A mixture of 2.5 mmol α-T3, 3.0 mmol of *N*-*t*-Boc-methylglycine hydrochloride, and 3.0 mmol of dicyclohexylcarbodiimide in dry pyridine (20 mL) were stirred at room temperature for 24 h. After evaporation in vacuo, the residue was triturated with ethyl acetate (50 mL), and dicyclohexylurea was removed by filtration. The filtrate was evaporated in vacuo, and the trituration was repeated twice. The *N*-*t*-Boc-methylglycine ester of α-T3 was isolated using flash chromatography (Flash 40+M silica gel columns, φ 40 × 150 mm, Biotage Japan Co., Ltd., Tokyo, Japan) and elution with 9:1 (*v*/*v*) n-hexane/ethyl acetate. Four mol/L HCl in dioxane was added to an acetone solution of *N*-*t*-Boc-methylglycine ester, and the mixture was stirred for 30 min. The solvent was evaporated in vacuo, and the residue was recrystallized from acetone to give the hydrochloride salt 2*R*-*α*-Tocotrienyl *N*-methylglycinate (α-T3MG). The product was a white solid.

α-T3MG: mp 170–173 °C; MS *m*/*z*: 496 (M-HCl + H^+^). ^1^H-NMR δ (CDCl_3_): α-tocotrienyl moiety 5.10 (3H, m, 3′,7′,11′,-H), 2.56 (2H, t, *J* = 7 Hz, 4-H_2_), 2.12–1.97 (19H, m, including 2.07 (3H, s, 7-CH_3_), 2.00 (3H, s, 8-CH_3_), 1.97 (3H, s, 5-CH_3_)), 1.78–1.52 (16H, m, including 1.60 (3H, s, 12′-CH_3_), 1.59 (6H, s, CCH_3_), 1.56 (3H, s, CCH_3_)), 1.23 (3H, s, 2-CH_3_), *N*-methylglycine moiety 9.95 (1H, s, NH), 4.09 (2H, s, -NCH_2_CO-), 2.80 (3H, s, -NCH_3_). Anal Calcd for C32H49NO3HCl + 0.5H_2_O: C, 71.02; H, 9.50; N, 2.59. Found: C, 71.00; H, 9.64; N, 2.58.

#### 4.4.2. R-α-Tocotrienyl *N*,*N*-Dimethylglycinate Hydrochloride

The synthesis procedure for 2*R*-*α*-Tocotrienyl *N*,*N*-dimethylglycinate hydrochloride (α-T3DMG) was the same as described in Section 4.4.1, except that *N*,*N*-dimethylglycine hydrochloride was added instead of *N*-*t*-Boc-methylglycine hydrochloride in the first reaction. The product was a white solid.

α-T3DMG: mp 186–188 °C; MS m/z: 510 (M-HCl + H^+^). ^1^H-NMR δ (CDCl_3_): α-tocotrienyl moiety 5.10 (3H, m, 3′,7′,11′-H), 2.64 (2H, t, *J* = 7 Hz, 4-H_2_), 2.15–1.93 (19H, m, including 2.11 (3H, s, 7-CH_3_), 2.04 (3H, s, 8-CH_3_), 2.01 (3H, s, 5-CH_3_)), 1.85–1.54 (16H, m, including 1.65 (3H, s, 12′-CH_3_), 1.58 (6H, s, CCH_3_), 1.56 (3H, s, CCH_3_)), 1.27 (3H, s, 2-CH_3_). *N*,*N*-dimethylglycine moiety 4.60 (2H, s, NCH_2_CO), 3.06 (6H, s, (CH_3_)_2_N). Anal. Calcd for C33H52NO3HCl + 0.2H_2_O: C, 72.09; H, 9.61; N, 2.55. Found: C, 72.05; H, 9.75; N, 2.53.

#### 4.4.3. R-α-Tocotrienyl Acid Succinate

The amount of 2.7 mmol of succinic anhydride and dimethylaminopyridine were added to dry dichloromethane containing α-T3 (2.5 mmol). The reaction mixture was stirred at room temperature for 20 h, and the solvent was evaporated in vacuo. The residue was treated with 100 mL water and acidified with 1 mol/L HCl. The solution was then extracted three times with 50 mL ethyl acetate. The organic layer was dried over anhydrous sodium sulfate and evaporated. The residue was fractionated using flash chromatography (Flash 40 + M silica gel columns, φ 40 × 150 mm, Biotage Japan Co., Ltd.) and eluted with 8:2 (*v*/*v*) n-hexane/ethyl acetate (8:2, *v*/*v*). The obtained compound, 2*R*-α-Tocotrienyl acid succinate (α-T3Suc), was a colorless oil.

α-T3Suc: MS *m*/*z*: 524 (M^+^). ^1^H-NMR δ (CDCl_3_): α-tocotrienyl moiety 5.12 (3H, m, 3′,7′,11′,-H), 2.59 (2H, m, 4-H_2_), 2.13–1.95 (19H, m, including 2.09 (3H, s, 7-CH_3_), 2.01 (3H, s, 8-CH_3_), 1.96 (3H, s, 5-CH_3_)), 1.82–1.59 (16H, m, including 1.67 (3H, s), 1.60 (6H, s), 1.59 (3H, s)), 1.25 (3H, s, 2-CH_3_). succinyl moiety 2.92 (2H, t, *j* = 7 Hz) 2.82 (2H, t, *j* = 7 Hz). Anal. Calcd for C33H48O5: C, 75.53; H, 9.22. Found: C, 75.32; H, 9.26.

### 4.5. Water Solubility

The aqueous solubility of each ester was determined by adding 50 μmol of each compound to 1 mL of distilled water in amber test tubes and incubating the tubes at 25 ± 1 °C in a constant-temperature water bath. The test tubes were shaken for 3 h, and the suspensions were filtered using a membrane filter (MF-Millipore™ Membrane Filter, 0.45 µm pore size, Merck KGaA, Darmstadt, Germany). The filtered media, including α-T3DMG, was assayed using the liquid chromatography (LC)-MS/MS method described in Section 4.8.

### 4.6. Enzymatic Hydrolysis of α-T3 Derivatives

The hydrolysis of esters was analyzed at 37 °C in phosphate-buffered saline (PBS) containing commercially available rat liver microsomes (In Vitro Technologies, Inc., Baltimore, MD, USA). The microsomes (20 mg/mL) were adjusted to a protein concentration of 0.11 mg/mL and preincubated at 37 °C for 5 min before adding the esters.

Stock solutions of the esters were prepared in a 5% methanol aqueous solution. Enzymatic reactions were initiated by adding 50 µL ester stock solution (final concentration 50–800 μmol/L) and 50 μL PBS to 900 µL preheated reaction medium containing rat liver microsomes in amber test tubes. These reactions were incubated at 37 °C; and, at various times, 100 μL aliquots were removed and mixed with 350 µL ethanol. Samples were then vortexed for 2 min and centrifuged at 1750 g for 5 min. The aliquot was analyzed using the LC-MS/MS system described in Section 4.8. The initial hydrolytic rate (μmol/L α-T3 formed per min) was calculated from the initial slope of the plot of α-T3 concentration versus time.

The effects of eserine on α-T3DMG hydrolysis in rat liver microsomes were also examined using similar methods, with 50 µL eserine aqueous solution instead of PBS, added to the mixtures with liver microsomes. Eserine was tested at 0–2.0 mmol/L.

### 4.7. Micellization of α-T3DMG with Taurocholic Acid

#### 4.7.1. Preparation of Aqueous Solutions of α-T3DMG with Taurocholic Acid

A stock solution of α-T3DMG was prepared in milliQ and combined with different concentrations of taurocholic acid (TCA) aqueous solution to the final molar ratios (α-T3DMG/TCA = 1/0.1–4). The compositions of the solutions are listed in Table 4. The fitting curves were obtained by the plateau model using GraphPad Prism to indicate a smoothing curve connecting each point.

#### 4.7.2. Determination of α-T3DMG in n-Octanol and Water Phases

The prepared solutions described in Section 4.7.1 were combined with the same volume of n-octanol and then vortexed for 2 min. The solutions were then centrifuged at 20,600× *g* for 20 min. The upper layer (n-octanol) and the lower layer (water) were collected, diluted with ethanol, and subjected to LC-MS/MS, as described in Section 4.7.

#### 4.7.3. Determination of Particle Size Distribution and Z-Potential

The z-average with PDI and the z-potential of the aqueous solution of α-T3DMG with or without TCA, as described in Section 4.7.1, were determined using a Zetasizer Nano ZS (MALVERN, Worcestershire, UK). Measurements were independently performed three times for each sample.

#### 4.7.4. pH Measurement

The pH of the aqueous solution of α-T3DMG with or without TCA is described in Section 4.7.1. It was measured using a LAQUAtwin pH-11 pocket water quality meter (HORIBA, Ltd., Kyoto, Japan).

### 4.8. LC-MS/MS Analysis

The LC-MS/MS analysis was performed using an LCMS-8050 and a Shimadzu UFLC system (Shimadzu, Kyoto, Japan) with a CAPCELL PAK C18 UG120 column (3 μm, 2.0 mm × 100 mm, Shiseido, Tokyo, Japan). The mobile flow was a binary gradient: (A) water and (B) 0.1% acetic acid in methanol containing 10 mmol/L ammonium acetate. The total flow rate was 0.4 mL/min; 0 min at 80% B, 2 min at 80% B, 4 min at 100% B, 8 min at 100% B, and 8.1 min at 80% B. The column temperature was 40°C. The mass spectrometer was equipped with electrospray ionization and operated in positive ion mode. Identification and quantitation were based on the MS/MS multiple reaction monitoring mode using the transition ion as follows: m/z 425.30 → 165.00, [M + H]^+^ α-T3 adduct; *m*/*z* 510.20 → 58.05, and [M+H]^+^ α-T3DMG adduct.

### 4.9. Dosing Protocol

#### 4.9.1. Preparation of Bile Duct-Ligated Rat Model

The SD rats were fasted for 16 h, and they were provided drinking water ad libitum prior to the administration of the test compounds. The rats were administered three types of mixed anesthetic agents (medetomidine, midazolam, and butorphanol) under isoflurane anesthesia. To avoid the influence of intrinsic bile flow on intestinal absorption, a catheter was inserted into the bile ducts of the rats. The catheter was ligated with strings, and intrinsic bile flow was excluded. This animal model was referred to in an animal experimental book in Japanese, titled in English as, “An atlas of experimental animal technology” published in 1998; this method is very similar to that described in Figure 5 of Jaber et al. [23].

#### 4.9.2. Preparation of Dosing Solution and Gastrostomic Administration

Dosing solutions were prepared as follows: (i) 5.88 mmol/L α-T3DMG and 5.88 mmol/L TCA in water (α-T3DMG:TCA = 1:1), (ii) 5.88 mmol/L α-T3DMG and 11.76 mmol/L TCA in water (α-T3DMG:TCA = 1:2), and (iii) 5.88 mmol/L α-T3 and 11.76 mmol/L TCA in water (α-T3:TCA = 1:2). The α-T3 was dispersed in silica powder in ethanol, and the solvent was removed in vacuo prior to dispersion in water. The ligated rats were administered 29.4 mmol/kg of α-T3DMG or α-T3 via gastrostomy. The doses were 12.5 mg/kg of body weight equivalent for α-T3.

#### 4.9.3. Blood Sampling

Blood samples (200 µL) were collected from the external jugular vein under isoflurane anesthesia using heparinized syringes at 0.5, 1, 2, 3, 4, 6, 8, and 24 h. Plasma was isolated by centrifugation, combined with an equal volume of ethanol three times the volume of n-hexane, vortexed for 2 min, and centrifuged at 1750× *g* for 10 min. The organic layer was evaporated using N_2_ gas. The residue was reconstituted with 100 µL of ethanol, sonicated for 10 s, and subjected to LC-MS/MS, as described in Section 4.8.

### 4.10. Statistical Analysis

Statistical significance was determined using a one-tailed unpaired *t*-test; a *p* < 0.05 was considered significant. Data were analyzed using GraphPad Prism 6 (GraphPad Software).

## Figures and Tables

**Table 1:** Kinetic parameters for hydrolysis of α-T3DMG, α-T3MG, and α-T3Suc in rat plasma and liver microsome at pH 7.4 and 37 °C.

Compound	*K*_m_ (×10^−3^ mol·L^−1^)	*V*_max_ (×10^−6^ mol·L^−1^·min^−1^)	*V*_max_/*K*_m_ (×10^−3^ min^−1^)
Rat plasma			
α-T3MG	1.460	0.5573	0.3817
α-T3DMG	0.6641	0.08138	0.1225
α-T3Suc	1.291	0.1033	0.08002
Rat liver microsome			
α-T3MG	0.1765	2.051	11.62
α-T3DMG	5.723	139.5	24.38
α-T3Suc	0.06162	0.07426	1.205

The values are obtained from a Michaelis–Menten curve fitting (GraphPad Prism).

**Table 2:** Physicochemical property of the particles in the aqueous solutions of α-T3DMG with or without TCA.

Molar Ratio of TCA vs. α-T3DMG (X).	logX	pH	Z-Average (nm)	PDI	Z-Potential (mV)
5.88 mmol/L α-T3DMG					
0	-	3.0	282.0 ± 8.9	0.542 ± 0.034	95.4 ± 2.5
0.3	−0.52	3.2	12.6 ± 0.2	0.428 ± 0.014	48.3 ± 7.5
0.9	−0.05	3.8	3.8 ± 1.1	0.228 ± 0.039	19.1 ± 12.2
1	0	3.8	5.3 ± 3.5	0.203 ± 0.064	8.70 ± 6.17
1.5	0.18	4.1	3.1 ± 0.0	0.161 ± 0.045	−4.35 ± 1.99
3	0.48	4.6	4.3 ± 1.8	0.217 ± 0.009	−0.0299 ± 0.260
4	0.60	4.7	163.7 ± 84.5 ^a^	0.466 ± 0.068	−22.1 ± 3.37
23.52 mmol/L α-T3DMG					
0	-	2.7	307.8 ± 8.6	0.490 ± 0.020	no data
0.3	−0.52	3.5	6.6 ± 0.1	0.336 ± 0.026	
0.9	−0.05	3.9	4.2 ± 0.4 ^b^	0.286 ± 0.231 ^a^	
1	0	3.9	3185 ± 327 ^b^	0.250 ± 0.179 ^a^	
1.5	0.18	4.4	9.2 ± 6.1	0.190 ± 0.045	
3	0.48	4.8	22.4 ± 15.1	0.219 ± 0.051	
4	0.60	4.8	23.7 ± 12.2	0.217 ± 0.005	

^a^ Ambiguous data because all the observed peaks of diameter are <100 nm and the test solutions are completely transparent. ^b^ Ambiguous data due to occurring precipitation of the component as shown in Figure 3B.

**Table 3:** Pharmacokinetic parameters for α-T3 after gastrostomy administration of α-T3DMG in bile duct-ligated rats.

Parameter	α-T3DMG:TCA = 1:1 (logX = 0)	α-T3DMG:TCA = 1:2 (logX = 0.3)
*C*_max_ (μmol/L)	0.432 ± 0.150	0.913 ± 0.289
*T*_max_ (h)	6	8
*AUC*_0–24h_ (nmol·L^−1^·h)	5.615 ± 1.055 *	10.477 ± 3.789 *
*MRT* (h)	9.40 ± 0.46	12.8 ± 6.18

Values are presented as mean ± SD. The X value indicates TCA molar ratio of TCA to α-T3DMG. * *p* < 0.05; unpaired *t*-test.

**Table 4:** Composition of the mixture solutions containing α-T3DMG with or without TCA.

α-T3DMG (mmol/L)	TCA Concentration (mmol/L)																			
2.94	0	0.29	0.59	0.88	1.18	1.47	1.76	2.06	2.35	2.65	2.94	3.23	3.53	3.82	4.12	4.41	4.70	5.88	8.82	11.8
5.88	0	0.59	1.18	1.76	2.35	2.94	3.53	4.12	4.70	5.29	5.88	6.47	7.06	7.64	8.23	8.82	9.41	11.8	17.6	23.5
11.8	0	1.18	2.35	3.53	4.70	5.88	7.06	8.23	9.41	10.6	11.8	12.9	14.1	15.3	16.5	17.6	18.8	23.5	35.3	47.0
23.5	0	2.35	4.70	7.06	9.41	11.8	14.1	16.5	18.8	21.2	23.5	25.9	28.2	30.6	32.9	35.3	37.6	40.0	70.6	94.1
	Molar ratio of TCA vs α-T3DMG (: X)																			
	0	0.1	0.2	0.3	0.4	0.5	0.6	0.7	0.8	0.9	1	1.1	1.2	1.3	1.4	1.5	1.6	2	3	4
	Log X																			
	-	−1.00	−0.70	−0.52	−0.40	−0.30	−0.22	−0.15	−0.10	−0.05	0	0.04	0.08	0.11	0.15	0.18	0.20	0.30	0.48	0.60

## Data Availability

Not applicable.

## Supplementary Materials

The following supporting information can be downloaded at https://www.mdpi.com/article/10.3390/molecules27092727/s1, Figure S1: Effect of eserine on hydrolysis of α-T3 ester derivatives in the rat liver microsome. Figure S2: Plasma α-T3 after administration of α-T3 with taurocholic acid.

## Sample Availability

Samples of the compound, α-T3DMG, are available from the authors.
