# Supplementary material for: Cationic N,N-Dimethylglycine Ester Prodrug of 2R-α-Tocotrienol Promotes Intestinal Absorption via Efficient Self-Micellization with Intrinsic Bile Acid Anion
Source: Molecules. 2022 Apr 23;27(9):2727. doi: 10.3390/molecules27092727 (PMC9102404; doi:10.3390/molecules27092727)
Supplement: Supplementary file 1 [file molecules-27-02727-s001.zip › molecules-1689557-supplementary.pdf]

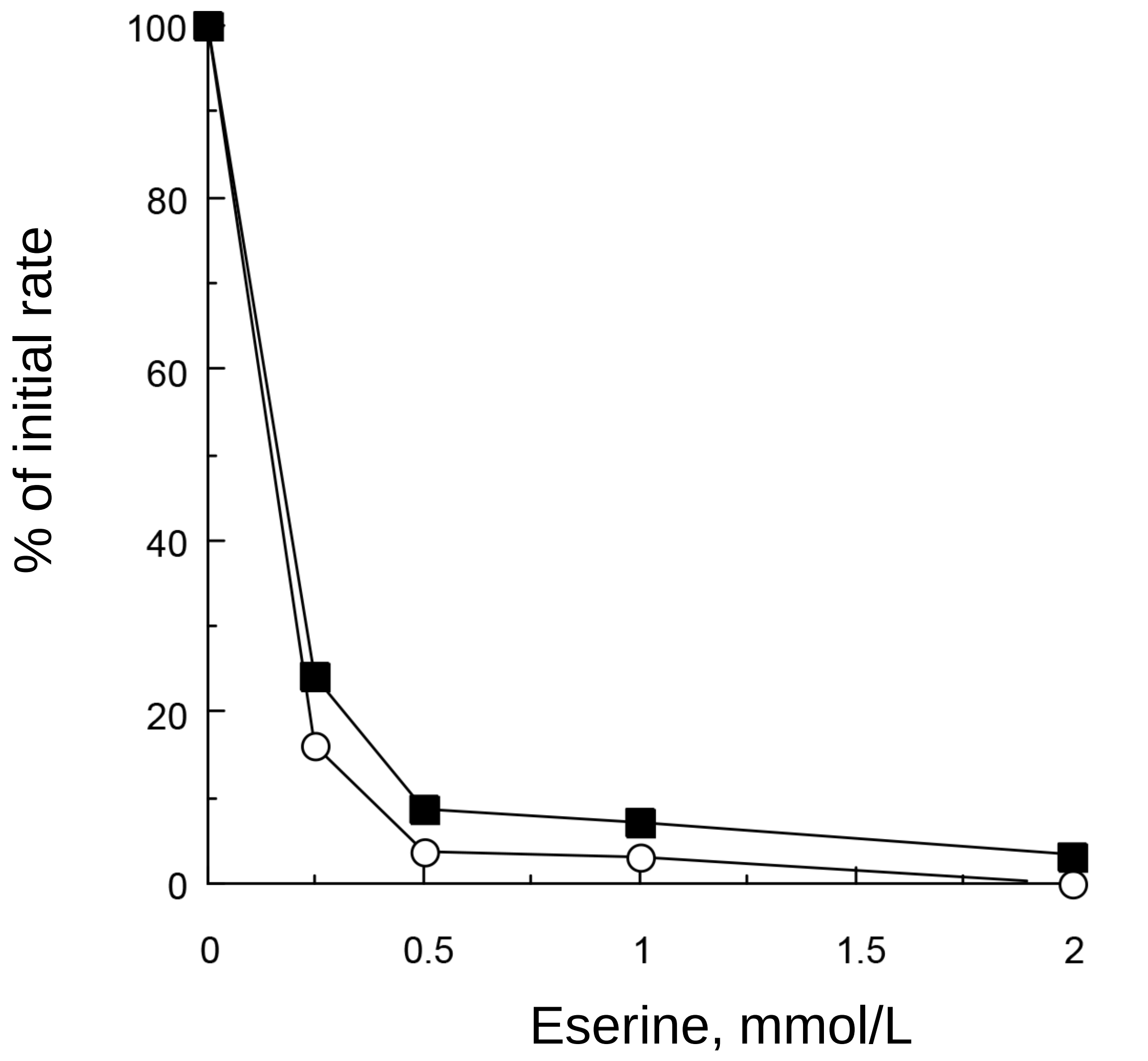

Supplementary Figure S1. Effect of eserine on hydrolysis of α-T3 ester derivatives in the rat liver microsome.
Key; (○) α-T3DMG, (■) α-T3MG

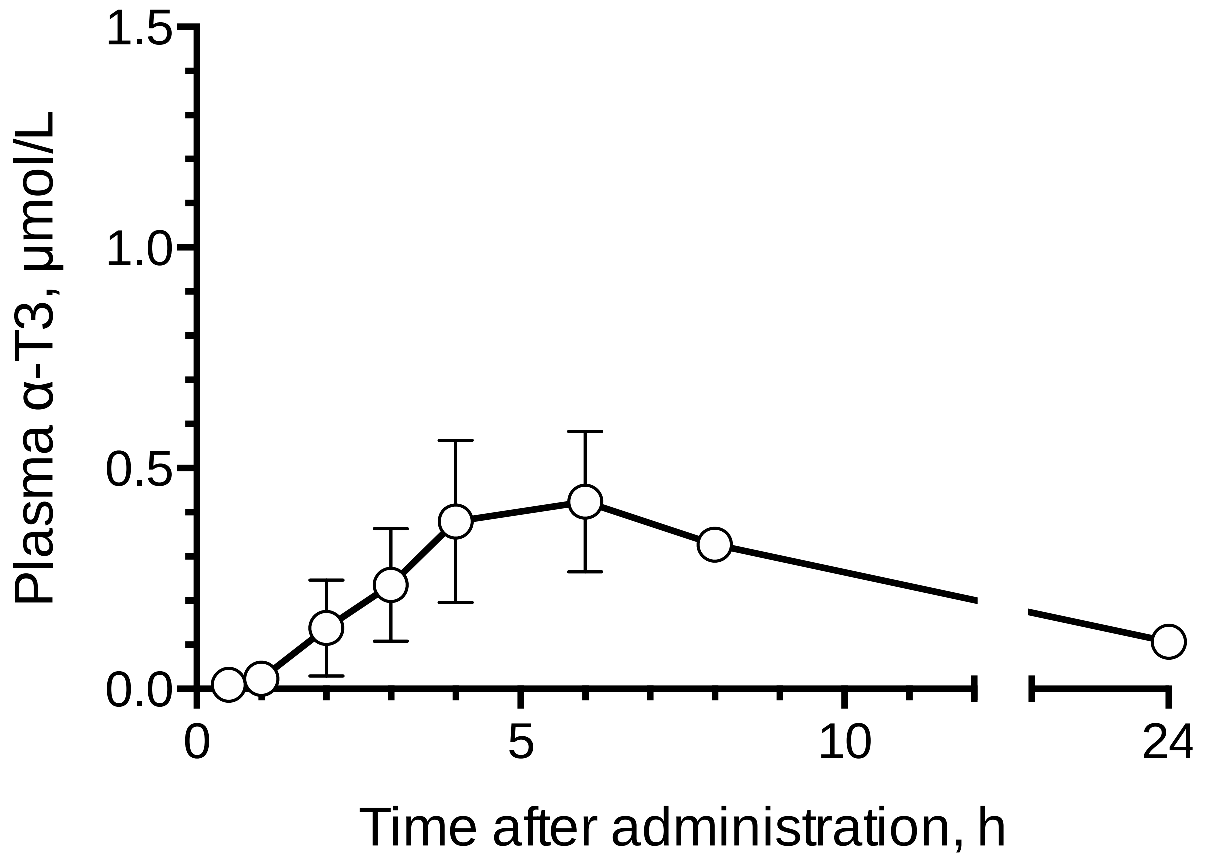

Supplementary Figure S2. Plasma α-T3 after administration of α-T3 with taurocholic acid.
